# Visualizing tephra deposits and sedimentary processes in the marine environment: The potential of X‐ray microtomography

**DOI:** 10.1002/2015GC006073

**Published:** 2015-12-30

**Authors:** Adam J. Griggs, Siwan M. Davies, Peter M. Abbott, Mark Coleman, Adrian P. Palmer, Tine L. Rasmussen, Richard Johnston

**Affiliations:** ^1^ Department of GeographyCollege of Science, Swansea UniversitySwanseaUK; ^2^ Advanced Imaging of Materials FacilityCollege of Engineering, Swansea UniversitySwanseaUK; ^3^ Centre for Quaternary Research, Department of GeographyRoyal HollowayEghamUK; ^4^ Centre for Arctic Gas Hydrate, Environment and ClimateUniversity of TromsøTrømsoNorway

**Keywords:** tephra, X‐ray microtomography, micromorphology, bioturbation, paleoceaonography

## Abstract

Localized tephra deposition in marine sequences is the product of many complex primary and secondary depositional processes. These can significantly influence the potential applicability of tephra deposits as isochronous marker horizons and current techniques, used in isolation, may be insufficient to fully unravel these processes. Here we demonstrate the innovative application of X‐ray microtomography (µCT) to successfully identify tephra deposits preserved within marine sediments and use these parameters to reconstruct their internal three‐dimensional structure. Three‐dimensional visualizations and animations of tephra dispersal in the sediment permit a more thorough assessment of postdepositional processes revealing a number of complex microsedimentological features that are not revealed by conventional methods. These features include bioturbation burrows and horizontally discontinuous tephra packages, which have important ramifications for the stratigraphic placement of the isochron in a sedimentary sequence. Our results demonstrate the potential for utilizing rigorous two and three‐dimensional microsedimentological analysis of the ichnofabric to enhance and support the use of tephra deposits as isochronous marker horizons and to identify the stratigraphic position that best reflects the primary fallout of ash. The application also provides an exceptional insight into the style and rate of sedimentation processes and permits an assessment of the stratigraphic integrity of a tephra deposit. We discuss the possibility of applying these µCT methods to the identification of cryptotephras within various paleoclimatic sequences and to enhance our understanding of marine sedimentation processes.

## Key Points

- Novel application of µCT to visualize marine tephra deposits in 3‐D
- The 3‐D visualizations and animations enhance assessment of postdepositional processes
- Rapidity of tephra deposition may determine ensuing bioturbation activity in the oceans

## 1 Introduction

### 1.1 Marine Tephrochronology: Understanding Tephra Deposition and Associated Sedimentary Processes

Tephra (vitreous volcanic ejecta) deposits have been identified in a number of disparate sedimentary contexts and when traced between sequences, act as time‐synchronous marker horizons to establish independent and precise tie points between paleoenvironmental records [e.g., *Haflidason et al*., 2000; *Lowe et al*., 2008; *Lowe*, 2011]. Establishing precise and independent correlations between marine, terrestrial, and ice‐core records is essential for determining if leads and lags occur in the climatic system [e.g., *Austin and Hibbert*, 2012; *Davies et al*., 2012]. However, in order to fully exploit these tie‐lines, it is necessary to thoroughly assess the stratigraphical and depositional integrity of the tephra horizons. This is particularly crucial in the marine realm due to the complexity of processes that transport tephra to the water surface and within the ocean system [e.g., *Austin et al*., 2004; *Brendryen et al*., 2010; *Abbott et al*., 2013; *Griggs et al*., 2014; *Davies et al*., 2014].

The fundamental prerequisite of tephrochronology is that, following an eruption, tephra is deposited and preserved rapidly into a sequence and its stratigraphic position relates to the timing of the event [*Lowe*, 2011]. Tephra deposits in the marine environment, however, are particularly vulnerable to secondary transport and reworking processes such as bioturbation or sediment loading [e.g., *Ruddiman and Glover*, 1972]. These processes may displace or blur the lower contact of the tephra horizon by either drawing material upward and/or downward through the profile, decreasing the concentration of the peak in glass shards [*Ruddiman and Glover*, 1972; *McCave*, 1988; *Bromley*, 1996; *Todd et al*., 2014; *Cassidy et al*., 2014]. Positioning the tephra isochron, therefore, may be problematic. Nonetheless, the susceptibility of tephra‐derived glass particles to mobilization gives rise to excellent tracers for assessing the degree of vertical mixing within a marine record [e.g., *Ruddiman and Glover*, 1972]. Given that age models and a variety of proxy information are derived from foraminifera deposited within the sediment, the potential blurring caused by biological mixing mechanisms can affect the stratigraphic integrity of the tephra as well as the overall proxy record [*McCave*, 1988; *Thomson et al*., 1995].

Tephra‐derived glass shard concentration profiles in combination with the identification of ice‐rafted detritus (IRD), grain‐size characteristics, and the degree of geochemical homogeneity are some of the features that can help to improve our understanding of the effect of secondary processes on tephra deposits [e.g., *Lackschewitz and Wallrabe‐Adams*, 1997; *Brendryen et al*., 2010; *Abbott et al*., 2011, 2013, 2014; *Gudmundsdóttir et al*., 2011; *Griggs et al*., 2014; *Davies et al*., 2014]. Macroscale bioturbation structures can also be revealed by 2‐D visual observations of the core [e.g., *Ruddiman and Glover*, 1972; *McCave et al*., 1995]. Moderate bioturbation can be detected by distinctive patterns on the core surface, and, in some instances, reworking may become so extensive that it visually homogenizes the tephra within the host sediment [*Manville and Wilson*, 2004; *Cassidy et al*., 2014]. X‐radiography has also been employed to reveal the preservation of burrows within thick tephra deposits in a number of marine cores near New Zealand, and in the NE Atlantic following a Heinrich event [*McCave et al*., 1995].

It has become increasingly apparent, however, that the complexities of the processes operating on tephra deposits cannot be fully unraveled using these existing stratigraphic techniques in isolation [*Griggs et al*., 2014]. This is particularly problematic for cryptotephra deposits in which small glass shards occur in exceptionally low concentrations and so are highly susceptible to these secondary depositional processes [*Abbott et al*., 2011]. Any stratigraphic displacement of a deposit may lead to chronological uncertainties and erroneous use of tie points, which has important ramifications for establishing the relative timing of climatic signals preserved within paleosequences [*Abbott et al*., 2013; *Davies et al*., 2014].

Recent investigations have demonstrated the potential of applying three‐dimensional (3‐D) techniques such as X‐ray microtomography (µCT) to visualize microsedimentological features within Quaternary sediments [*Kilfeather and van der Meer*, 2008; *Tarplee et al*., 2011; *Bendle et al*., 2015]. The ability to examine the 3‐D internal architecture of tephra deposits within cores may permit the visualization of a range of sedimentary features and facilitate the examination of the sediment/tephra interface through several planes of the core. This visualization has the potential to improve the stratigraphic accuracy of the tephra isochron, while also supporting the interpretation of marine sedimentary processes. Thus, this paper aims to establish if, in the first instance, tephra deposits can be distinguished from enclosing sediments using µCT. The results of this experimentation are then used to reevaluate two marine tephra horizons from the North Atlantic, bringing together 2‐D and 3‐D analyses to accurately establish the position of the isochron. Finally, the paper considers the applicability of µCT in understanding the biological mixing mechanisms that are inherent within the marine environment.

### 1.2 X‐Ray Microtomography (µCT): Principles and Geological Applications

High‐resolution µCT is a nondestructive method for imaging internal structures in 3‐D at micronscale spatial resolution, based upon the fundamental principle that X‐ray attenuation is a function of X‐ray energy and the density and atomic number (Z) of the material being scanned [*Landis and Keane*, 2010; *Cnudde and Boone*, 2013]. A series of radiographs are compiled to create 3‐D representations that can be computationally manipulated to perform a wide array of visualizations [*Ketcham and Carlson*, 2001]. Successful geological applications of µCT include the study of the pore geometry of carbonate reservoirs [*Purcell et al*., 2009], rock fluid analysis [*Wennberg et al*., 2009], glacigenic deformation structures [*Tarplee et al*., 2011], and proximal volcanic textures [*Polacci et al*., 2006]. As glass shards and their host sedimentological matrix fall within known density ranges [*Turney*, 1998; *Blockley et al*., 2005], they should exhibit different X‐ray absorption properties, allowing the detection and isolation of such materials in a marine core through the use of µCT techniques. However, the contrast between these materials is very small, with quartz (2.65 g/cm^3^) and heavy minerals exhibiting similar densities to wholly basaltic glass (>2.5 g/cm^3^). These similarities may cause an overlap between the sediment and tephra‐phase reconstructions but, if this contrast can be detected, it should be possible to isolate the two phases using histograms of density variations. This study pinpoints the µCT instrumental parameters required and develops a protocol to successfully differentiate tephra, manifested as glass shards in the study, from enclosing sedimentary material. With this new protocol, the 3‐D structure of two Faroe Marine Ash Zones (FMAZ) from North Atlantic marine core JM11‐19PC was examined. JM11‐19PC was selected as an experimental test bed for this work because of the excellent preservation of visible tephra deposits, which were previously analyzed using thin‐section analysis and 2‐D techniques [*Griggs et al*., 2014].

### 1.3 The Faroe Marine Ash Zones

The Faroe Marine Ash Zones II and IV (FMAZ II, IV) are Marine Isotope Stage (MIS) 3 basaltic tephra deposits that are commonly identified within marine sediment cores in the Faroes region [e.g., *Rasmussen et al*., 2003; *Wastegård et al*., 2006; *Wastegård and Rasmussen*, 2014], including the JM11‐19PC core outlined in *Griggs et al*. [2014]. The high concentration of glass shards and the visibility of the tephra horizons in the core are ideal for testing the applicability of µCT. Gradational shard concentration profiles derived from standard tephra counting suggest vertical mixing may have been operating, although no clear bioturbation structures are apparent in thin section [*Griggs et al*., 2014].

Both FMAZ II and FMAZ IV, preserved in JM11‐19PC, have been interpreted by *Griggs et al*. [2014] as primary fall deposits on the basis of strong geochemical homogeneity, limited IRD input, and a low concentration of coarse‐grained shards. For FMAZ II, 2‐D micromorphological investigations revealed discrete packages of tephra as glass shards beneath the main horizon, thought to be a consequence of postdepositional sedimentary loading. Combined with a peak in the shard concentration profile, *Griggs et al*. [2014] placed the isochron at 304–305 cm. For FMAZ IV, the stratigraphical placement of the isochron coincided with the highest glass shard concentration determined by optical microscopy techniques at 542–543 cm. Combining 3‐D imaging with 2‐D micromorphology and shard concentration profiles allows an assessment of the stratigraphic position of each tephra isochron.

## 2 Materials and Methods

### 2.1 Core Processing and Sedimentology

**Figure 1:**
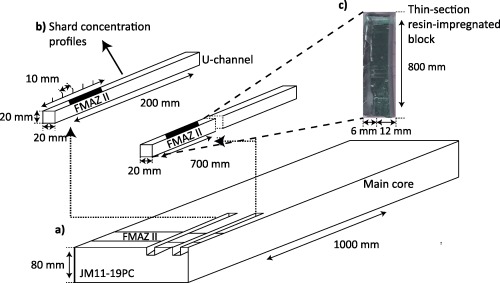
Schematic summary of the U‐channel sampling from marine core JM11‐19PC. (a) Two U‐channels, with the dimensions 20 mm × 20 mm × 200 mm extracted parallel to each other from the archived core for (b) quantification of shard concentrations and (c) production of a resin‐impregnated block for thin‐section and tomographic analyses.

The JM11‐19PC core was retrieved from 1179 m water depth on the central North Faroe Slope in the southeastern Norwegian Sea (62°48′98″N, 03°52′04″E) [see *Griggs et al*., 2014]. Two parallel U‐channels with a 20 mm by 20 mm cross‐sectional area were extracted from core sections spanning FMAZ II and IV (Figure 1). It has been confirmed, through glass shard concentration profiles, that they span the onset of deposition, the visible expression, and the overlying decline in shard concentration of the two ash zones. One U‐channel was used for quantifying the glass shard content of the sequence (Figure 1b) and extracting suitable shards for geochemical analysis, as outlined in *Griggs et al*. [2014]. The second U‐channel (Figure 1c) was used for preparing thin sections following procedures outlined in *Griggs et al*. [2014]. The resin‐impregnated blocks produced for the thin‐section work were then scanned using the µCT system.

### 2.2 Determining µCT Parameters for Tephra Detection

#### 2.2.1 µCT Instrumentation

**Figure 2:**
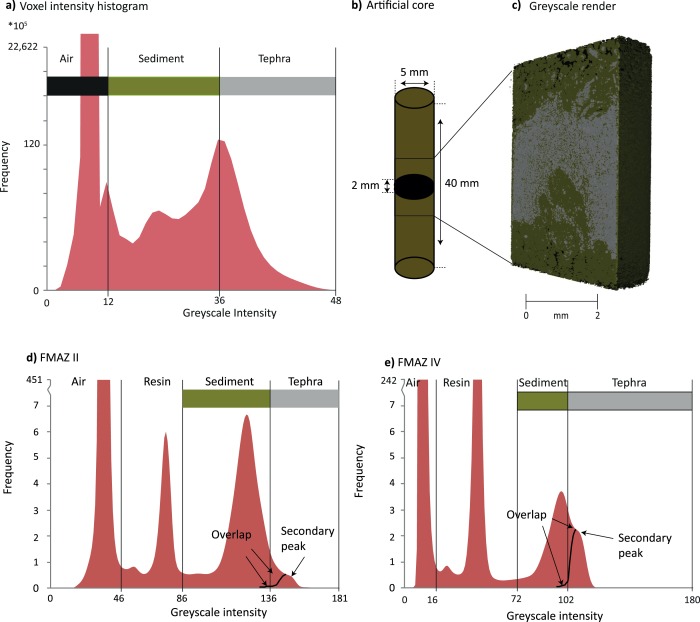
Example of the µCT procedure used to identify basaltic glass shards in a straw and in the resin‐impregnated blocks. (a) Voxel‐intensity histogram for the straw. The vertical lines denote the thresholds defined by the histogram to isolate air, sediment, and tephra. (b) Schematic representation of the artificially created straw. (c) Three‐dimensional grey scale render of the straw. Grey = basaltic glass shards (representing tephra); green = sediment. (d and e) Voxel intensity histograms generated by the tomographic analysis of the FMAZ II and FMAZ IV resin blocks, respectively. The additional intensity peaks in these examples are generated by the resin which was not a feature of the straw experiments. Vertical lines denote the defined thresholds to isolate air, resin, sediment, and glass (tephra).

To constrain the density contrast (expressed as grey scale intensity) between tephra and the host sediment, an artificial tephra deposit was created in a ∼20 mm length of plastic drinking straw of ∼5 mm internal diameter. A ∼2 mm thick layer of wholly basaltic glass shards (density >2.5 g/cm^3^) was added between two layers of freeze‐dried, marine sediment known to be devoid of tephra and composed of a mixture of heavy and light mineral grains (Figure 2b).

The basaltic‐filled straws were investigated using a Nikon XT H 225 microfocus X‐ray tomography system, with a 1.3 Megapixel Varian PaxScan 2520 amorphous silicon flat panel digital X‐ray imager, in reflection mode with a tungsten target. X‐ray tube voltages between 50 and 100 kV were trialed as these are similar to those used by *Ketcham and Carlson* [2001] to distinguish between quartz and orthoclase. These minerals exhibit a small density contrast (2.65 versus 2.59 g/cm^3^) of a similar magnitude to the density contrast expected between basaltic glass and marine sediment. Low voltages are favored as photoelectric absorption is the dominant attenuation mechanism at energies <100 kV. The practical importance of this phenomenon is that photoelectric absorption is proportional to Z^4‐5^ (atomic number) of the attenuating material, and as a result, low energy X‐rays are more sensitive to differences in composition than higher energy ones [*Ketcham and Carlson*, 2001]. Scans revealing the most effective contrast were performed using a 50 kV X‐ray tube voltage, a current of 550 µA, with an exposure of 1000 ms, averaging 4 images per rotation step of 0.119°, resulting in 3016 images per scan and a voxel (3‐D pixel) size of 4.7 µm. The tomograms were reconstructed from 2‐D projections using a Nikon cone‐beam reconstruction algorithm and proprietary software (CTPro version 3.0, Nikon Metrology). The commercial software VGStudio Max 2.1.5 and free software Drishti [*Limaye*, 2012] were used to view the reconstructed data, 2‐D grey scale slices, and rendered 3‐D volumes.

#### 2.2.2 Delineating Tephra Using Voxel‐Intensity Histograms

The resulting µCT analysis of the basaltic glass‐filled straw was used to produce a histogram of voxel (3‐D pixel) intensities. This plots the frequency, or the number of voxel occurrences of a particular intensity, which are related to density, within the overall scan. The peaks allow us to qualitatively segment the image into different phases by placing thresholds between peaks [*Gonzalez and Woods*, 2007], which correspond to air, sediment, and glass (Figure 2a). In order to identify the threshold that differentiates between the tephra and sediment phases, we moved the threshold value until the tomographic render resembled the expected visual structure of the artificial straw, i.e., a lateral tephra horizon surrounded by sediment devoid of tephra (Figures 2b and 2c). Despite an overlap in the density ranges of basaltic glass and sediment, a distinct tephra deposit was successfully revealed when the threshold was placed at a grey scale intensity of 36 (Figures 2a and 2c). Grey scale intensities higher than this value are thought to equate to basaltic glass shards. The basaltic glass in Figure 2a has a higher absorption coefficient and/or contains a higher‐Z composition than the surrounding sediment, which is mainly composed of quartz. One limitation in defining instrument parameters, however, is the overlap between the sediment and tephra phase distributions that reflects a common problem with qualitative intensity‐based segmentation [see *Landis and Keane*, 2010] (Figure 2c). In Figure 2a, an overlap of grey scale intensities is apparent between high‐density sediment and lower‐density tephra/glass. Segmentation at the peak of the glass distribution (Figure 2a) excludes some lower‐density glass (occurring between 20 and 36 grey scale intensity) and this represents a minimum estimate of the total abundance of glass present. However, we expect the number of basaltic shards that exhibit lower grey scales to be exceptionally small. Placing the threshold at a grey scale intensity of 36 does not affect the overall structure of the tephra deposit because the grey scale frequencies with the greatest number of occurrences have been included.

The simulation experiment shows that tephra layers comprised of basaltic glass preserved within marine sediment can be successfully imaged with suitable experimental parameters on a µCT system. Defining thresholds, however, is a qualitative procedure and is specific to each scan. As a result, absolute thresholds that will distinguish between basaltic glass and sediment in a range of different depositional contexts and scans cannot be defined and uniformly applied. Our approach is to recommend optimal instrument parameters (see section 2.1) and validate the placement of thresholds with known areas of tephra either on core inspection or within thin sections.

### 2.3 Determining Basaltic Glass Grey Scale Thresholds in JM11‐19PC

**Figure 3:**
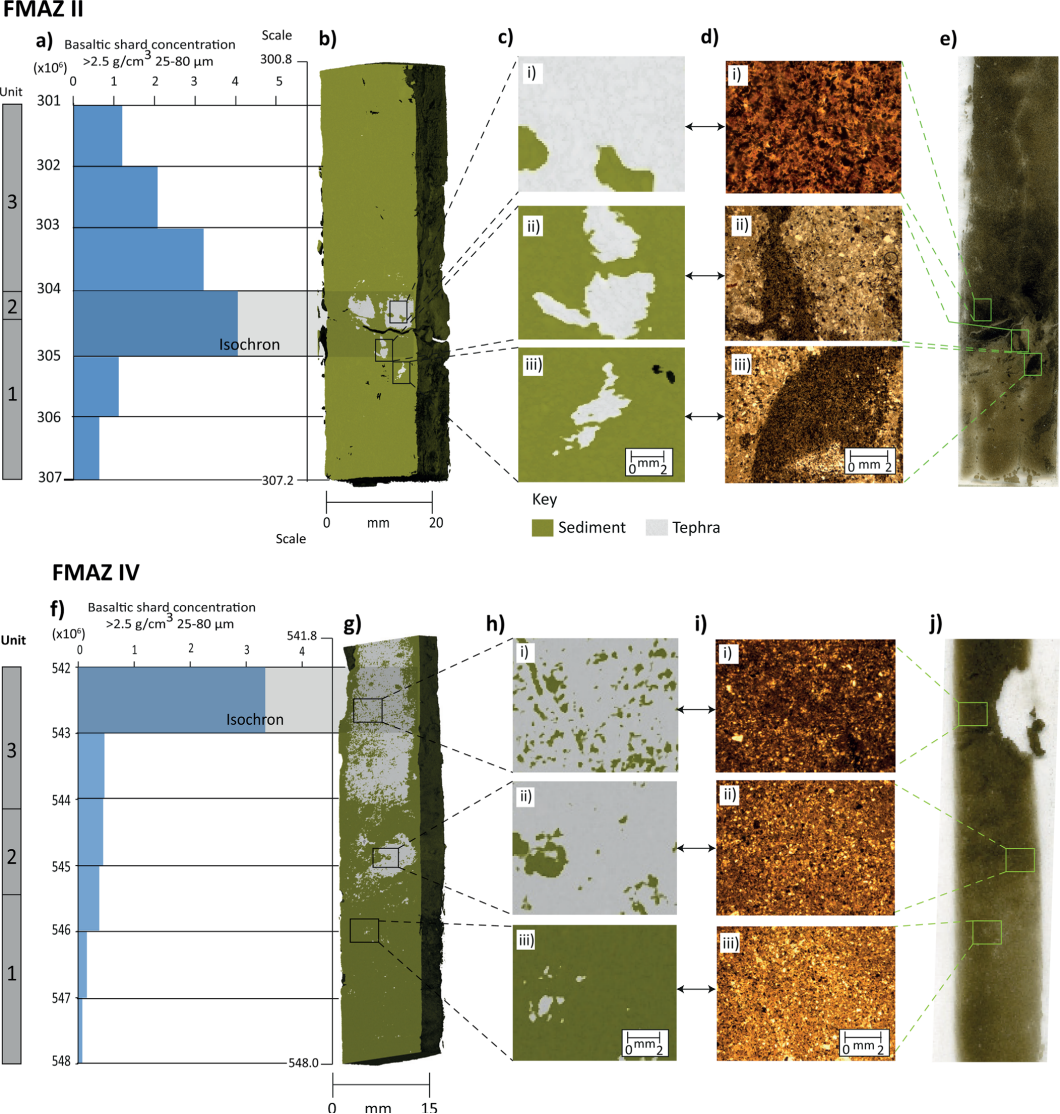
**FMAZ II:** composite thin‐section and µCT grey scale summary used to validate thresholds defined in Figure 2. (a′) Basaltic shard concentration profile (25–80 µm fraction). (b′) Grey scale 2‐D reconstruction from the surface of the resin block. Grey = basaltic glass shards (representing tephra); green = sediment. (c′) Indicative grey scale features presented in ×4 magnification aligned to (d′) indicative microfacies features derived from thin‐section analysis (×2.5 magnification). (e′) Thin‐section scan. c′ and d′ (i) Predominantly grey and structureless, aligned to massive, well‐sorted abundant glass shards. (ii) Typical of the second unit of the FMAZ II, composed of multiple packages and vertically oriented lens of glass shards (grey) in a sediment matrix (green). (iii) Isolated and vertically oriented package of glass shards within a coarse‐silt sediment matrix. **FMAZ IV:** composite thin‐section and µCT grey scale summary used to validate thresholds defined in Figure 2. (f) Basaltic shard concentration profile (25–80 µm fraction). (g) Grey scale 2‐D reconstruction from the surface of the resin block. Grey = basaltic glass shards (representing tephra); green = sediment. (h) Indicative grey scale features presented in ×4 magnification aligned to (i) indicative microfacies features derived from thin‐section analysis (×2.5 magnification). (j) Thin‐section scan. h and i (i) Predominantly grey with occasional packages of green dispersed randomly throughout, aligned to massive, moderately sorted, abundant glass shards. (ii) Moderate concentrations of glass shards (grey) within a moderately sorted coarse‐silt matrix (green). (iii) Occasional grey packages containing low concentrations of glass shards distributed randomly within a moderately sorted coarse‐silt matrix.

The intensity‐based segmentation procedure, outlined in section 2.2, was applied to the voxel intensity histograms generated for the FMAZ II and IV deposits preserved within resin‐impregnated blocks. Thin sections generated from these blocks were used to define the threshold values (Figures 2d and 2e). In comparison to the artificially created specimen, the intensity peaks for these cores are less pronounced and a distinct shoulder is observed at the falling limb of the sediment peak. The higher grey scale intensity values that form this shoulder are thought to represent areas of concentrated glass shards that are of a higher density relative to the surrounding sedimentary matrix. This interpretation was confirmed by comparing the grey scale 2‐D μCT slice from the surface of the resin‐impregnated block with the previously acquired 2‐D thin‐section image from the same surface (see Figure 3) and *Griggs et al*. [2014].

A striking similarity is revealed between the μCT‐derived glass shard distribution and the darker areas of concentrated glass shards seen in the thin section (Figure 3). For the FMAZ II, for example, the greatest concentration of glass, shown in grey, occurs at the subhorizontal, sharp contact at 304–304.5 cm (Figure 3c′i). A ×4 magnification of this structure seen in the thin section confirms the presence of a structureless deposit comprised of abundant and well‐sorted tephra shards (Figure 3d′i). In addition, below the main shard concentration peak, irregularly aligned packages of concentrated shards, surrounded by a coarse‐silt matrix and interpreted as lobate structures by *Griggs et al*. [2014], correspond with μCT‐identified glass‐rich areas (Figures 3c′ii, 3c′iii, 3d′ii, and 3d′iii). It is worth noting that the shape of the tephra lobes below the isochron exhibit a slightly different geometry from those observed in thin section and this may be because the thin section was cut from a slightly different angle and plane. The different configurations may also be due to the nature of the segmentation process, which may not completely separate glass shards in lower concentrations from the host marine sediment. For FMAZ IV, μCT reconstructions revealed tephra‐rich areas in the upper part of the profile which agree well with the thin‐section observations. As the glass shard concentrations decrease in the lower part of the profile, sporadic grey packages (tephra) are observed within a green‐shaded matrix in the 2‐D μCT render (Figures 3h′ and 3i′ii). A ×4 magnification of this feature in thin section confirms the presence of a coarse‐silt matrix with occasional well‐sorted pockets of glass shards (Figure 3i′ii).

The excellent agreement between the features observed in the thin sections and the 2‐D µCT‐derived reconstructions support the threshold definition and intensity‐based segmentation procedures. In our examples, we demonstrate that concentrated areas of glass shards are represented by a secondary peak/shoulder with higher grey scale intensity values. The prominence of this shoulder/secondary peak relative to the sediment phase is shown to vary between deposits due to contrasting shard/sediment concentrations and mineral compositions (Figure 2). Nonetheless, our visual confirmation approach indicates that the voxel intensity diagrams are appropriate for delimiting tephra and sediment and allow a 3‐D reconstruction of the tephra deposits, manifested here as basaltic glass shard concentrations.

## 3 Results

### 3.1 Three‐Dimensional Visualization of Tephra Structure

**Figure 4:**
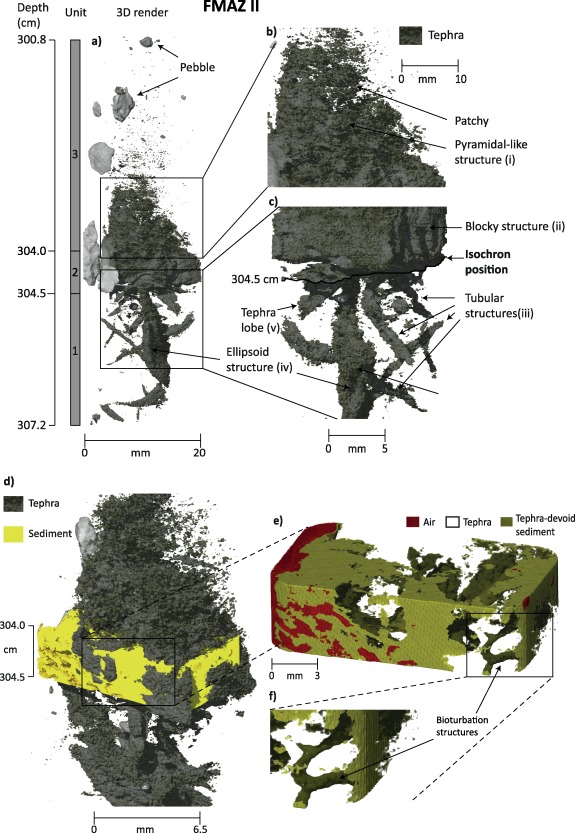
Three‐dimensional µCT render of the FMAZ II deposit (see supporting information Movies S1 and S2 for the animation). (a) Render of the whole resin block, displaying only the tephra deposit (pebbles are artefacts of the resin impregnation process). Structures have been magnified to highlight indicative sedimentological features in Figures 4b and 4c. The isochron position has also been highlighted. (d) Region of interest between 304 and 304.5 cm to show the presence of other intensity phases, i.e., sediment and air within this section. (e) Region of interest showing only sediment and air. (f) Magnification of Figure 4e to highlight bioturbation structures.

**Figure 5:**
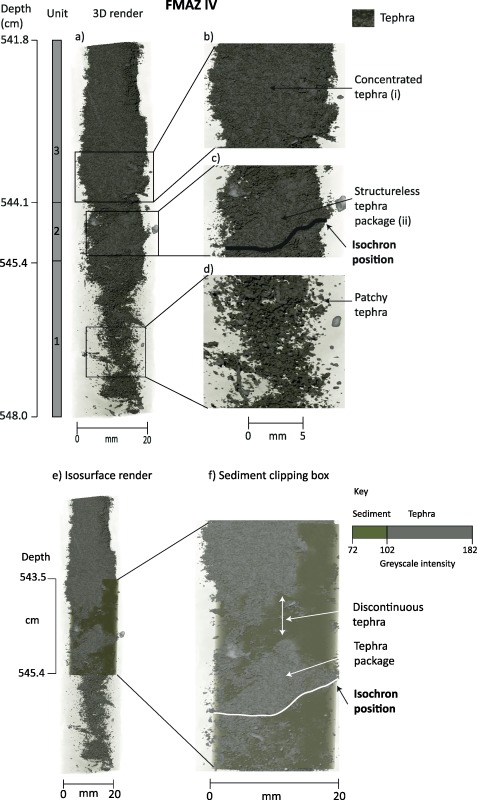
Three‐dimensional µCT isosurface render of the FMAZ IV deposit (see supporting information Movie S3 for the animation). (a) Render of the whole resin block, displaying only the tephra phase. Structures have been magnified to highlight indicative sedimentological features in Figures 5b–5d. The isochron position has also been highlighted and is defined according to features observed in Figure 6. (e) Render of the whole resin block, displaying the tephra phase with the addition of a region of interest between 543.5 and 545.4 cm which displays both sediment and tephra phases. (f) Magnification of the region of interest to illustrate the planar discontinuity between the tephra packages. The isochron position has also been illustrated.

Three‐dimensional visualizations and animations of each tephra deposit are presented in Figures 4 and 5 and in the supporting information Movies S1–S3. The ability to visualize and represent the complex 3‐D architecture of tephra within the U‐channel sample, distinguished from the surrounding sediment, provides an exceptional insight into the structure of tephra deposits and the sedimentary processes operating in the ocean. The nonuniform morphology of the deposits across three axes is presented, and the rotation allows the viewer to get a feeling for the depth of field within the sample and across the tephra/sediment interface.

#### 3.1.1 FMAZ II

The structure of this deposit is complex, and three units are defined according to distinct sedimentological features (Figure 4). The first occurs between ∼304.5 and 307.2 cm and consists of numerous tubes of high glass shard concentration that are orientated at different angles within the sediment. Unit 1 is shown in approximately the bottom third of the core section displayed in the supporting information Movie S1 and the lower portion of supporting information Movie S2, which displays this region‐of‐interest at a higher resolution. There is a large narrow ellipsoidal structure which is aligned vertically between ∼304.9 and 306.5 cm, with a width of ∼2 mm (Figure 4iv). In addition, a large tube is present that penetrates perpendicularly through the center of this ellipsoid structure (Figure 4iii). The largest tube is approximately ∼0.5 mm in width; ∼20 mm long and has a noticeable curvature with a shift in orientation at ∼304.9 cm.

The second unit occurs between ∼304.0 and 304.5 cm and consists of a distinct blocky structure (Figure 4d, supporting information Movie S1, and the top quarter of the section in supporting information Movie S2), representing a high concentration of glass, with minor undulations at the base (Figure 4ii). Despite the high concentration of glass within this blocky structure, there are a number of sediment‐filled burrows and voids penetrating through the deposit at numerous locations and angles with the best branched example having a subhorizontal repose (Figure 4f). Thin veneers of sediment are also seen draping this blocky tephra structure. These structures are observed by segmenting (threshold value defined in section 2.3) the tomographic data to digitally remove the tephra and visualize the sediment (Figure 4f).

The third unit occurs between ∼300.8 and 304.0 cm, comprising the top half of the section shown in supporting information Movie S1, and consists of a pyramidal‐like structure comprised mainly of tephra shards between 303 and 304 cm. Tephra is more concentrated on one side of the scanned block, sloping at a ∼60° incline (Figure 4i). The concentration of tephra declines rapidly above 303 cm with a loss of structure observed by ∼303.2 cm. The rest of this unit is characterized by sporadic and low concentration glass packages.

#### 3.1.2 FMAZ IV

**Figure 6:**
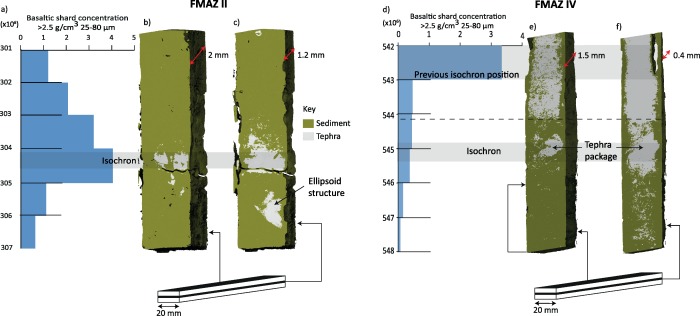
Three‐dimension visualization of different clipping planes reveal subsurface structure of the FMAZ II and FMAZ IV deposits (i.e., (b and e) face and (c and f) middle plane of the resin blocks). (a and d) Basaltic shard concentration profiles (25–80 µm fraction).

Three units are defined within this deposit. The first occurs between ∼545.4 and 548.0 cm, spanning approximately the bottom third of the section represented in supporting information Movie S3, and is characterized by discontinuous tephra packages that vary in concentration and appear across all planes of the core (Figure 5d). The overall appearance of this unit is a dispersed zone of glass shards. The second unit, between ∼544.1 and 545.4 cm, consists of a large structureless, but concentrated unit of tephra, although the concentration is variable through the different planes of the 3‐D render (Figures 5f and 6 and the middle section of supporting information Movie S3). A diffuse and nonhorizontal contact is observed between unit two and the underlying sediment of unit 1 (Figure 5a). There is also a narrow band of sediment (∼10 mm) separating unit two from the highly concentrated and structureless tephra deposit in unit three ∼541.8–544.1 cm (Figures 5e and 5f) and can be observed as a diagonal band with a low concentration of glass shards in supporting information Movie S3.

## 4 Discussion

### 4.1 Using 3‐D Visualization to Reinterpret Depositional Processes

#### 4.1.1 FMAZ II: Sedimentation Processes

The 3‐D reconstruction and visualizations of sedimentary structures associated with the FMAZ II requires a reinterpretation of the dominant depositional processes. Based on thin‐section analysis, *Griggs et al*. [2014] reported the presence of sedimentary loading features below the main deposit which strongly suggested rapid tephra deposition. This new 3‐D reconstruction, represented in supporting information Movies S1 and S2, highlights the presence of bioturbation structures that were not apparent in the initial investigation of thin sections. Lobes and oval structures were noted below the main tephra deposit in thin section but it is only from the 3‐D μCT reconstruction that we can now see that they represent a 2‐D slice through some of these tubes. Both lobate (Figure 4v) and bioturbation structures (Figure 4iii) are seen within unit one and we believe that the former represent the loading structures seen in thin section. We postulate that the rapidity of deposition caused some destabilization of the sediment water interface at the seabed, which permitted the movement of glass shards into the underlying sediment, forming these lobate structures (Figure 4v).

The bioturbation burrows reveal the mixing of tephra through sediment and vice versa (Figures 4e and 4f). This mutual mixing suggests that bioturbation may have been active during tephra deposition as *Bromley* [1996] suggests that burrows become filled with material that differs from the surrounding sediment in most endobenthic communities. We suggest that tephra deposition was rapid and as a consequence, the bioturbation mixing layer shifted upward at a similar rate. This would result in the preservation of burrows, as seen in unit one and supporting information Movie S2, as the sediment below the tephra layer was quickly isolated from the influence of bioturbation. Ash deposits >1 cm thick, similar to the FMAZ II, typically smother the benthos [*Carter et al*., 1995] and *McCave et al*. [1995] noted that a consequence of rapid ash deposition is the enhanced preservation of burrows beneath an ash horizon. This mechanism supports our interpretation that the blocky structure was deposited rapidly (Figure 4). It is, of course, possible that the burrowing features may have been superimposed on the sediment fabric following postdepositional recolonization, but there is no strong evidence to support this scenario.

No visible burrows are evident within unit three. Given the gradual decline in shard concentration in this unit (Figure 3a) [*Griggs et al*., 2014], it is likely that bioturbation reestablished, which homogenized sediment above the blocky structure. The absence of preserved burrows may reflect a higher bioturbation rate resulting in the complete homogenization of the tephra and sediment due to a slowdown in the upward migration of the mixing layer after tephra deposition. It is also possible that additional postdepositional processes such as reworking by currents may have altered the ichnofabric of the sediment and prevented the preservation of burrows. This possibility emphasizes the potential for combining a range of tephrostratigraphic and visualization techniques to fully unravel the sedimentary history of the deposits.

#### 4.1.2 Isochron Placement

The 3‐D reconstruction of this deposit supports the isochron placement of *Griggs et al*. [2014] and provides a more comprehensive analysis of the processes operating at the time. The high concentration of glass shards in unit two between ∼304 and 304.5 cm (the midpoint of the original isochron) has a dense appearance in all planes and a sharp contact with the underlying material suggests rapid influx of a high tephra volume. This 3‐D reconstruction confirms that the position at ∼304.5 cm equates to the correct stratigraphical placement of the isochron (Figure 4c).

#### 4.1.3 FMAZ IV: Sedimentation Processes

A key contrast between this deposit and FMAZ II is the absence of bioturbation burrows (Figure 5 and supporting information Movie S3). This absence may be a result of enhanced bioturbative activity and slow upward migration of the bioturbation mixing layer. We suggest that bioturbation may have completely homogenized the sediment and destroyed any diagnostic sedimentological features such as tubular burrows. Deposition of this ash zone occurred during Dansgaard Oeschger (DO) interstadial 12, which may have provided favorable conditions for a diverse range of ocean‐dwelling organisms [*Wastegård and Rasmussen*, 2014]. The increase in fauna may have been an additional factor that promoted increased bioturbative activity [*Rasmussen et al*., 1996]. Furthermore, in comparison to the FMAZ II, there are no distinct loading structures, indicating a slower rate of tephra deposition. This feature points toward a more gradual tephra delivery and fewer disturbances to the sedimentary system. Thus, bioturbative disturbance may be enhanced in core sections where there is relatively uniform sedimentation. Such uniformity may have important ramifications for the movement of microorganisms, which are commonly utilized as proxies, within stratigraphic intervals exhibiting uniform sedimentation conditions. Grey scale renders of the FMAZ IV suggest there is a significant amount of sedimentary matrix within the tephra deposit (green shading in Figure 6e and reduced tephra concentrations in supporting information Movie S3), which also supports a slower rate of tephra deposition.

#### 4.1.4 Isochron Placement

**Figure 7:**
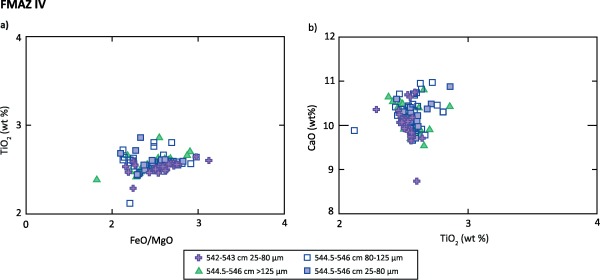
Major oxide results (wt %) for glass shards extracted from JM11‐19PC 544.5–546 cm (unit 1) and 542–543 cm (unit 3). The JM11‐19PC 542–543 cm (FMAZ IV) compositions are derived from shard analyses obtained from the 25–80 µm fraction investigated in *Griggs et al*. [2014]. Only the 25–80 µm fraction is shown as other grain sizes are identical in composition. Data have been normalized to 100% total oxide concentrations, and the full data set is available in the supporting information S1–S3.

The 3‐D evidence from this deposit suggests the operation of more complex sedimentological processes than those deduced by *Griggs et al*. [2014] and that the isochron position should be realigned accordingly. The concentrated tephra packages seen in unit two of the µCT‐derived reconstruction (Figure 5) are not observed in thin section or in the glass shard concentration profile (Figure 3f) which highlights lateral variability within this core. This tephra package in unit two may represent the initial input of ash into the sequence as a result of primary fallout and subsequent deposition through the water column. However, it is puzzling that the highest concentration of glass shards as determined by optical microscopy (Figure 3f) and depicted in the µCT‐derived reconstruction (Figures 5, 6 and supporting information Movie S3) is seen in unit three. Unit three may reflect subsequent input of tephra from bottom current redistribution during a period of intensified deep water formation [*Ezat et al*., 2014]. Major element analysis indicates that the shards from unit two and three have identical geochemical signatures (Figure 7) and no correlative events have been found in the tephra record for the Greenland ice cores [*Bourne et al*., 2015]. Thus, it is not possible to determine whether this is a reflection of upward mixing of a deposit from a single eruption, an amalgamation of tephra deposits from several closely spaced eruptions or continuous tephra delivery by oceanic processes to the core site. Based on our current observations, we suggest that the placement of the isochron should occur at the initial primary influx of tephra at the nonhorizontal contact seen between ∼544.9 and 545.3 cm (Figures 5f and 6). This realignment highlights the ability of employing 3‐D reconstruction to aid correct stratigraphical placement of the isochron.

### 4.2 Recommendations for Future Work

Our 3‐D visualization of tephra deposits, in the form of basaltic glass shards, has provided an exceptional insight into sedimentation processes within the marine environment and highlighted the structures and distribution of tephra material within a sedimentary record. The microstructural features observed highlight the complexity and variability of shard concentration within a sedimentary core. On the one hand, the uneven distribution of tephra in a core section is a cause for concern when seeking isochrons, but conversely, this visualization tool can be used to enhance our confidence in the stratigraphic placement of isochrons. What is more this technique may prove valuable for distinguishing between primary and reworked deposits (cf. X‐ray density scanning method outlined by *Hopkins et al*. [2015]).

In relation to the FMAZ II, bioturbation was clearly an active process, but the overall picture indicates limited impact on the position of the tephra isochron, marked by a thick, densely packed deposit, due to rapid deposition. In the same way, we propose that vertical mixing has had very little impact on the integrity of other proxies around this horizon. These observations would be difficult without this 3‐D visualization. No direct evidence for bioturbation processes was observed in the down‐core shard concentration profiles and thin‐section analysis. These findings demonstrate that these techniques will not always fully capture the complexity of tephra distribution observed in the 3‐D visualizations, but in this context, the isochron and its stratigraphic position are intact.

For the FMAZ IV, lateral variability across the core was a prominent feature that only became evident from the 3‐D visualizations. During extraction of sediment for shard counting, a small subsample of ∼1 g is taken from one side of a core. The internal lateral variability observed from the µCT scan clearly highlights that this type of sampling may not capture and/or overrepresent areas of concentrated glass shard packages, which may produce a biased concentration profile and potentially affect the correct stratigraphical placement of the isochron (Figure 6). Such lateral variability and biased sampling may explain the change required in locating the position of the FMAZ IV isochron.

Given the spatial complexity and variability observed within the small resin block presented here, multiple subsamples across several parallel vertical profiles may better constrain shard concentrations and the lateral and horizontal continuity of a horizon. This is potentially important for cryptotephra deposits that exhibit a diffuse distribution and lower concentration peaks of <100 shards. Where higher glass shard concentrations permit the use of µCT analysis the technique can be used to corroborate the position of the isochron. However, for the successful application of µCT analysis, thin sections are also crucial to permit an assessment of (a) composition, grading, and sorting of sediments and glass shards; (b) contacts between layers; (c) microfabrics and textures to determine any imposed stresses to the sediment; and (d) mineralogy [*van der Meer and Menzies*, 2011; *Bendle et al*., 2015]. These analyses represent important diagnostic sedimentological information that provide crucial building blocks for interpreting depositional processes that cannot be obtained using µCT alone. Furthermore, each time a new core is investigated (due to the changes in mineralogical composition and thus attenuation differences), it is necessary to create a thin section for grey scale threshold validation, i.e., to check that segmentation corresponds with glass shards. Therefore, the integration of micromorphological analysis and µCT offers a novel approach to understanding the microsedimentological characteristics of tephra deposits.

While we have successfully employed µCT to examine high‐concentration glass shard deposits with a visible expression, it is unclear whether it can be used to visualize tephra deposits in low concentrations (i.e., cryptotephras). It may be possible to capture cryptotephras by exploring host sediments, e.g., peat, that exhibit a significantly different absorption coefficient to glass shards. This may increase the attenuation contrasts, thus reducing overlap between tephra and host sediments, and make segmentation more viable. In addition, this study has solely focused on visualizing basaltic glass shards that have a small density contrast with marine sediments. Less dense rhyolitic material may show a greater density contrast with the host sediment which could aid intensity segmentation.

If the size of the scanned block is reduced, or if the scan is more focused on a section of the block, it may be possible to increase the scanning resolution of the deposit, permitting the employment of edge detection, gradients, or local variance to provide a more robust method of phase segmentation [*Landis and Keane*, 2010; *Bendle et al*., 2015]. Indeed, it is likely that targeted scanning of specific intervals within sequences would increase the µCT detection limits suitable for cryptotephra identification as the proportion of glass shards relative to sediment should increase and will improve the segmentation procedure.

## 5 Conclusions

1. Preliminary experimental work on a simulated tephra layer demonstrates the potential for successfully isolating tephra deposits from marine sediments using µCT analysis. Thin‐section images of two ash deposits in a North Atlantic marine core are employed to visually ensure the correct placement of thresholds to successfully isolate tephra and sediment phases.
2. The resultant 3‐D visualization of the FMAZ II highlights the presence of a large blocky structure which is used to constrain the stratigraphical placement of the isochron. The preservation of underlying bioturbation burrows is thought to be indicative of a catastrophic tephra input event. The absence of burrows for the FMAZ IV has been linked to enhanced levels of bioturbation and prolonged tephra input.
3. The 3‐D reconstructions provide additional evidence for deciphering the multitude of processes operating in the marine environment and ensure the correct utilization of marine tephra as an isochronous marker horizon. Future µCT applications may aid the identification of ichnofauna in marine environments based upon the structure and ichnological fabric of the tubular burrows.
4. Future experimentation is necessary to extend the detection limits and applicability of µCT for the visualization of cryptotephra deposits in marine, lake, and terrestrial environments.

## Supporting information

Movie S1

Movie S2

Movie S3

Data Set S1

Supporting Information S1
