# Supplementary material for: Visualizing tephra deposits and sedimentary processes in the marine environment: The potential of X‐ray microtomography
Source: Geochem Geophys Geosyst. 2015 Dec 30;16(12):4329–43. doi: 10.1002/2015GC006073 (PMC4951705; doi:10.1002/2015GC006073)
Supplement: Supplementary file 5 — Supporting Information S1 [file GGGE-16-4329-s005.docx]

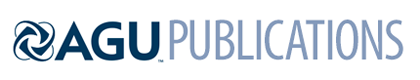


*Geochemistry, Geophysics, Geosystems*

Supporting Information for

**Visualising tephra deposition and associated sedimentary processes in the marine environment: the potential of X-ray microtomography**

Adam J. Griggs^a^ , Siwan M. Davies^a^ , Peter M. Abbott^a^ , Mark Coleman^b^ , Adrian P. Palmer^c^, Tine L. Rasmussen^d^ , Richard Johnston^b^

^a^Department of Geography, College of Science, Swansea University, Singleton Park, Swansea, SA2 8PP, UK.

^b^ Advanced Imaging of Materials (AIM) Facility, College of Engineering, Swansea University Bay Campus, Fabian Way, Crymlyn Burrows, Swansea, SA1 8EN, UK. ^c^Centre for Quaternary Research, Department of Geography, Royal Holloway, Egham Hill, Egham, TW20 0EX, UK.

Centre for Arctic Gas Hydrate, Environment and Climate, University of Trømso, NO9037, Trømso, Norway.

**Introduction**

The supplementary data reports major element geochemical analyses from individual shards from 545-546 cm (FMAZ IV) in JM11-19PC. This is used to determine whether shards from this depth exhibit the same geochemical properties as those reported in 542-543 cm (Griggs et al., 2015). Secondary standards of Lipari and BCR2G have also been reported.

The supplementary animations provide an aid for visualising the dispersal of glass-shards enclosed within marine core JM11-19PC for both the FMAZ II and FMAZ IV deposits

**Additional Supporting Information (Files uploaded separately)**

Data set S1: Raw geochemical results of 545-546 cm

Data set S2: Geochemical results for the Lipari standard recorded during analysis of 545-546 cm

Data set S3: Geochemical results for the BCR2G standard recorded during analysis of 545-546 cm

Movie S1: Animation of the FMAZ II enclosed within JM11-19PC

Movie S2: Animation of the FMAZ II enclosed within JM11-19PC. Higher resolution region-of-interest focussed on the tephra horizon and bioturbation features.

Movie S3: Animation of the FMAZ IV enclosed within JM11-19PC
